# Advocacy for Change: An Osteopathic Review of Traumatic Brain Injury Among Combat Veterans

**DOI:** 10.7759/cureus.25051

**Published:** 2022-05-16

**Authors:** Gehan A Pendlebury, Peter Oro, William Haynes, Thomas R Byrnes, James Keane, Leonard Goldstein

**Affiliations:** 1 Dermatology, Nova Southeastern University Dr. Kiran C. Patel College of Osteopathic Medicine, Fort Lauderdale, USA; 2 Neurology, A.T. Still University School of Osteopathic Medicine in Arizona, Mesa, USA; 3 Radiology, A.T. Still University School of Osteopathic Medicine in Arizona, Mesa, USA; 4 Osteopathy, A.T. Still University School of Osteopathic Medicine in Arizona, Mesa, USA; 5 Internal Medicine, A.T. Still University School of Osteopathic Medicine in Arizona, Mesa, USA; 6 Clinical Education Development, A.T. Still University School of Osteopathic Medicine in Arizona, Mesa, USA

**Keywords:** veterans health administration (vha), mild traumatic brain injury, moderate traumatic brain injury, veterans health, post traumatic stress disorder (ptsd), traumatic brain injury, combat veterans, mild head injury, closed head injury, military trauma

## Abstract

As a "signature injury" of the Iraq and Afghanistan wars, traumatic brain injury (TBI) remains a major health concern among military service members. Traumatic brain injury is associated with a wide range of symptoms which may be cognitive, emotional, psychological, biochemical, and social in nature. Mild TBI (mTBI) ranks as the most common traumatic brain injury among veterans. Due to the absence of specific symptoms, mTBI diagnosis may be challenging in acute settings. Repetitive traumatic brain injury during combat deployments can lead to devastating chronic neurodegenerative diseases and other major life disruptions. Many cases of TBI remain undetected in veterans and may lead to long-term adverse comorbidities such as post-traumatic stress disorder (PTSD), suicide, alcohol disorders, psychiatric diagnoses, and service-related somatic dysfunctions. Veterans with TBI are almost twice as likely to die from suicide in comparison to veterans without a history of TBI. Veterans diagnosed with TBI experience significant comorbid conditions and thus advocacy for improved care is justified and necessary. Given the complexity and variation in the symptomatology of TBI, a personalized, multimodal approach is warranted in the evaluation and treatment of veterans with TBI and other associated conditions. As such, this review provides a broad overview of treatment options, with an emphasis on advocacy and osteopathic integration in the standard of care for veterans.

## Introduction and background

Traumatic Brain Injury (TBI) is defined as either a mechanical (blunt trauma) or biomechanical (blast injury) force sufficient to result in a neural insult to the brain. The psychological and physical consequences of the trauma can be devastating to affected individuals and their families. The United States Department of Defense (DoD) defines TBI as “…a traumatically induced structural injury and/or physiological disruption of brain function as a result of an external force that is indicated by new onset or worsening of at least one of the following clinical signs, immediately following the event: (1) Any period of loss or a decreased level of consciousness, (2) Any loss of memory for events immediately before or after the injury, (3) Any alteration in mental state at the time of the injury (e.g., confusion, disorientation, slowed thinking), (4) Neurological deficits (e.g., weakness, balance disturbance, praxis, paresis/plegia, changes in vision, other sensory alterations, aphasia), (5) Intracranial abnormalities (e.g., contusions, diffuse axonal injury, hemorrhages, aneurysms)” [1].

Furthermore, the DoD classifies TBI into the following categories based on severity: mild, moderate, and severe. Table 1 displays TBI categorization based on the severity of symptoms.

**Table 1:** Classification of traumatic brain injury (TBI) Table 1 is adapted from the United States Department of Veterans Affairs (VA) and Department of Defense (DoD) Clinical Practice Guidelines for the Management and Rehabilitation of Post-Acute Mild Traumatic Brain Injury, Version 3.0 [2].  If the patient meets the criteria in more than one level of severity (mild/moderate/severe), a higher level is assigned. ^a^Alteration of consciousness or mental status must be directly related to the causative injury. Typical symptoms include feeling dazed, experiencing an unawareness of surroundings, difficulty thinking clearly, difficulty responding appropriately to mental status questions, amnesia surrounding the injury, and/or confusion. ^b^The DoD recommends against using the Glasgow coma scale (GCS) scores to diagnose TBI. However, the GCS is used by other organizations such as the American College of Surgeons (ACS). The ACS recommends using the individual components of the GCS (i.e., E4V4M5) rather than the sum score [2,3].

	Mild	Moderate	Severe
Structural Imaging	Normal	Normal or abnormal	Normal or abnormal
Loss of consciousness	0-30 min	>30 min and <24 hours	>24 hours
Alteration of consciousness or mental state^a^	Up to 24 hours	>24 hours	>24 hours
Post-traumatic amnesia	0-1 day	>1 and <7 days	>7 days
Glasgow coma scale^b^	13–15	9–12	<9

Combat veterans are among the highest at-risk populations to develop TBI and significantly associated comorbidities such as post-traumatic stress disorder (PTSD), suicide, and service-related somatic dysfunctions [4]. The DoD statistical data from 2000 to 2020 report 434,618 TBI cases (of all severities) among military personnel [5]. Cases of mild TBI (mTBI) comprise 84.4% of the total and rank as the most common traumatic brain injury. Of all recorded military TBI cases, the United States Army has the highest prevalence at 58.5% of the total compared to other branches of the military [5]. The most common mechanisms of injury in combat veterans involve blasts followed by an object hitting the head, a head hitting an object, falls, vehicular accidents or crashes, and other service-related injuries [4]. Blast overpressure without a primary or secondary impact may also induce TBI.

Traumatic brain injury has been coined a "signature injury" of the Iraq and Afghanistan wars. Veterans who screen positive for TBI are at a much higher risk (up to 80%) for comorbid psychiatric diagnoses, such as PTSD, anxiety, and depressive disorders [4]. Almost half of all veterans with combat-related mTBI qualify for PTSD diagnosis. Likewise, approximately one-third with mTBI experience depression, suicidal ideation, suicide attempts, and suicide completion. Veterans with a TBI diagnosis are 1.55 times more likely to die from suicide in comparison to veterans without a TBI diagnosis [4]. Further life impacts include alcohol-related disorders, pain conditions, unemployment, and severe mental health disorders (mood/anxiety disorders) [4]. Traumatic brain injuries present a higher risk of perilous comorbidities and therefore warrant further advocacy to improve the standard of care and overall quality of life for veterans. 

Healthcare providers utilize VA/DoD treatment and diagnostic guidelines as the gold standard to treat veterans with acute or chronic TBI and their associated complications [2]. These guidelines have improved the detection and prognosis of patients who suffer from TBI. However, osteopathic manipulative treatment (OMT) has not been incorporated in VA/DoD guidelines as a potential treatment for combat veterans. Osteopathic manipulative treatment is effective in relieving pain in numerous settings including TBI, spinal cord trauma, neck trauma, and postoperatively [6-8].

Pathophysiology of traumatic brain injury: A brief overview

The pathomechanism of TBI involves the disruption of the anatomical and physiological relationships of interconnected brain structures. Understanding these pathways is crucial for devising appropriate treatments. The etiologies of TBI are vast and include, but are not limited to, falls, sports injuries, combat-related injuries, and bodily collisions. Various brain regions may be injured during these events, which makes diagnosing TBI challenging due to the diverse and nonspecific clinical presentations [9].

The TBI-related injuries can be classified as either primary or secondary injuries [10]. Primary brain injuries result directly from an external mechanical force that is directed toward intracranial content. Secondary brain injuries, however, are sequelae of primary impact and can take minutes to days to cause deficits. These secondary mechanisms include, but are not limited to, diffuse inflammation in the brain, free radical generation due to mitochondrial dysfunction, neurotransmitter-mediated excitotoxicity, ischemia, and apoptosis [10].

Primary brain injury can lead to structural brain damage, extra-axial hematomas, focal or diffuse edema, and diffuse axonal injury (DAI). Extra-axial hematomas are subdivided into epidural hematoma (EDH) and subdural hematomas (SDH) [10]. An EDH is commonly caused by severe head trauma, most often to the temporoparietal region. This mechanism of injury commonly leads to rupture of the middle meningeal artery and hemorrhage into the epidural space. According to the Monro-Kellie doctrine, continued expansion of EDH can lead to brain herniation, while uncal herniation frequently causes occlusion of the oculomotor nerve [11]. Patients with EDH initially present with normal mental activity but might lose consciousness with severe EDH expansion [12]. Therefore, patients who present with clinical features of oculomotor nerve palsy after trauma should undergo immediate work-up for suspected EDH.

Like EDH, SDH can also manifest with signs of increased intracranial pressure. However, SDH is caused by a traumatic rupture of a bridging vein or veins. An EDH tends to occur in acute settings whereas SDH has a varying degree of presentation depending on the patient's age and the mechanism of injury [10]. In the acute setting of acceleration/deceleration injury, the surface of the brain shears against the undersurface of the skull, causing damage to a large area of the brain. For this reason, SDH is often considered more dangerous than EDH. Therefore, timely intervention may be necessary if symptomatic SDH is suspected in the context of trauma [10]. Subarachnoid hemorrhage (SAH) is another brain injury that may require urgent neurosurgical intervention and stabilization before any of the following should be considered.

Another consequence of severe primary brain injury includes diffuse axonal injury (DAI) [13]. Diffuse axonal injury has been strongly associated with a rotational or acceleration-deceleration head injury that damages the viscoelastic white matter axons [13,14]. Axon damage may result in accumulation of axonal transport products and axonal swelling. Such axonal damage may lead to Wallerian degeneration and formation of β-amyloid precursor protein (β-APP). This protein is linked to a plethora of neurodegenerative disorders. Additionally, injury to neurons (during DAI) results in cell death cascades which induce increases in cytotoxic intracytoplasmic calcium and reactive oxygen species [15]. Recent advances have shaped our understanding of brain changes following TBI. It has been demonstrated that TBI causes cellular membrane disruption and the subsequent release of damage-associated molecular patterns (DAMPs), which induce the production of inflammatory cytokines such as interleukin-1 (IL-1), IL-6, and tumor necrosis alpha (TNF-α). The release of TNF-α induces an inflammatory state in the brain, leading to increased cerebrospinal fluid and increased intracranial pressure [16]. In addition to the release of DAMPs following TBI, a massive release of glutamate from damaged neurons leads to neuronal excitotoxicity. Glutamate non-specifically binds to its receptors, N-methyl-d-aspartate (NMDA) and α-amino-3-hydroxy-5-methyl-4-isoxazolepropionic acid (AMPA), allowing for calcium influx into cells and eventual calcium-dependent degeneration [16]. Adenosine (a neuroprotective molecule) is released simultaneously with glutamate [17].

Neuroinflammation following TBI may have a protective property to the brain. Patients who received corticosteroids, minocycline (antibiotic with anti-inflammatory properties), or other immunosuppressive medication following the acute phase of TBI had deterioration of their condition [18]. Neuroinflammation during the acute phase of TBI improves debris clearance, maintenance of the blood-brain barrier, and immune regulation, among other benefits. Therefore, inhibiting these processes can worsen TBI symptoms [18].

The central nervous system (CNS) has a documented waste clearance system which has been termed the glymphatic system [19]. The glymphatic system consists of fluid channels that line the dura and meningeal arteries. Within the system is astroglia which supplies neurons with nutrients such as glucose, amino acids, and lipids. The glymphatic system eliminates toxic metabolites such as beta-amyloid (Aβ) and phosphorylated tau (p-tau). The glymphatic system functions predominantly during sleep and its function is reduced during wakefulness [20]. The function of the glymphatic system is mainly determined by the distribution and density of aquaporin-4 channels in astrocytes. Post-TBI neuroinflammation and other TBI-related symptoms decrease the function of astrocytes, which impairs the effectiveness of the glymphatic system [21]. Consequently, the accumulation of toxic metabolites contributes to persistent neurocognitive dysfunction [22].

This article provides an overview of the TBI pathophysiology and a deeper understanding of the mechanism of neurological injury. Combat veterans are a vulnerable population who experience higher rates of mTBI, making OMT a safe and efficacious treatment. Such viable solutions warrant further advocacy and research to improve the standard of care for veterans diagnosed with TBI. 

## Review

Clinical management: Current standard of care

As a broad diagnosis, treating TBI manifests complexities and intricacies. Traumatic brain injury is categorized in a clinical spectrum according to severity, ranging from acute trauma to long-term chronic sequelae. Therefore, no one-size-fits-all approach exists for the treatment of TBI. Each case is unique and deserves detailed attention in timely diagnosis. It is vital to implement an integrative, patient-centered approach tailored to the values, needs, experiences, and goals of the individual. Those with TBI may experience an array of symptoms, which have been grouped into the following clusters: cognitive dysfunction, neurobehavioral disorders, sensory disruption, somatic symptoms, and substance dependence [23]. These symptoms are outlined in Table 2. Physicians need to screen combat veterans for the presence of any of these symptoms and design a personalized treatment approach. A general overview of recommendations for the management and treatment of acute TBI is described in Table 3.

**Table 2:** Common neuropsychiatric sequelae of TBI TBI: Traumatic brain injury

Cognitive Dysfunction	Attention/Memory Deficits
Neurobehavioral Disorders	Post-traumatic stress disorder, aggressivity, impulsivity, suicide ideation, suicide attempts, suicide completion
Sensory Disruption	Visual changes, dizziness, hearing loss, altered smell perception, altered taste perception, hypersensitivity to touch
Somatic Symptoms	Chronic pain, headache, loss of libido, fatigue
Substance Dependence	Alcohol, non-dependent drug use, nicotine dependency

**Table 3:** Managing acute traumatic brain injury (TBI) a Signs and symptoms include progressively declining level of consciousness or neurologic exam, pupillary asymmetry, seizures, repeated vomiting, motor or sensory deficits, double vision, worsening headache, slurred speech, cannot recognize people, or disoriented to place [2]. b Military Acute Concussion Evaluation [2]. c Target values: Pulse Oximetry ≥ 95%; intracranial pressure (ICP) 20–25 mmHg; serum sodium 135–145; partial pressure of oxygen (PaO2 ) ≥ 100 mmHg;  brain tissue oxygen tension (PbtO2) ≥ 15 mmHg; International normalized ratio (INR) ≤ 1.4; partial pressure of carbon dioxide (PaCO2) 35-45 mmHg; cerebral perfusion pressure (CPP) ≥ 60 mmHg; platelets ≥ 75x103 /mm3; systolic blood pressure (SBP) ≥ 100 mmHg; temperature 36.0–38° C; hemoglobin ≥ 7 g/dl; pH 7.35–7.45; glucose 80–180 mg/dL [26]. d The DoD provides an algorithm for acute mTBI in the Clinical Practice Guidelines. Treatment for moderate/severe and penetrating brain injury (PBI) was adapted from the American College of Surgeons and the study by Kazim et al. [2,3,30].

TBI Severity	Management and Treatment
All	In cases of head trauma with loss of consciousness and/or post-traumatic amnesia, identify urgent/emergent signs and symptoms.^a^ When indicated, refer to neurosurgery if necessary. If non-emergent, evaluate injury for severity and follow appropriate guidelines. The Military Acute Concussion Evaluation 2 (MACE 2)^b^ scoring system may be used to track symptom progression and guide treatment [2].
Mild TBI (mTBI)	Restrict activity and brain stimulation. Observe and monitor patient for 24 hours. Monitor for signs of deterioration^a^. If patient deteriorates, obtain computerized tomography (CT) scan without contrast, and refer to neurosurgery depending on imaging results [24]. Provide education about mild TBI and secondary injury prevention [2].
Moderate/Severe TBI (sTBI)^d^	Follow Advanced Trauma Life Support (ATLS) guidelines to ensure adequate airway, breathing, circulation, and cervical spine immobilization. The Brain Trauma Foundation provides comprehensive recommendations for the treatment of acute severe traumatic brain injury [25]. Clinical management to reduce secondary brain injury^c^: manage oxygen and carbon dioxide levels; maintain normal systolic blood pressure; adjust the temperature, glucose, hemoglobin, and electrolyte levels (especially sodium) [26].
Penetrating TBI (pTBI)^d^	Treatment is highly dependent on the pathophysiology of injury; head CT scan and/or cerebral angiography should inform treatment [27]. Recent data demonstrate improved morbidity and mortality with aggressive broad-spectrum antibiotic prophylaxis, in combination with avoidance of debridement [28]. Prophylactic anticonvulsants may also be beneficial [29].

Long-term conventional treatments for mTBI symptoms

Many long-term symptoms are associated with mTBI. Due to the complex nature of TBI, each patient may present with multiple symptoms and overlapping comorbidities. To effectively address TBI symptoms and sequelae, the VA/DoD recommends a symptom-based, individualized treatment approach to improve symptoms and regain function [5].

The VA/DoD guidelines group TBI sequelae into physical, cognitive, and behavioral/emotional symptoms. Physical symptoms include headache, dizziness/vertigo, balance problems, nausea, fatigue, sleep or visual disturbance, sensitivity to light, hearing difficulties or loss of hearing, tinnitus, and sensitivity to noise. Cognitive symptoms include problems with attention, concentration, memory, speed of processing, judgment, executive functions, speech and language, and visual-spatial function. Behavioral/emotional symptoms include depression, anxiety, agitation, irritability, impulsivity, and aggression. Conventional treatments for these sequelae integrate patient education, psychotherapeutic modalities, pharmacological modalities, or a combination of the three. The VA/DoD guidelines target all sequelae domains. Key VA/DoD guideline takeaways are described in Table 4.

**Table 4:** Conventional treatments for mTBI symptoms ^a^ Detailed guidelines for the management of headaches [46]. ^b^ Detailed guidelines for the management of chronic insomnia disorder [47]. ^c^ Visual disturbances include sensitivity to light, difficulty focusing, and blurry vision. ^d^ Medications associated with visual symptoms include antihistamines, anticholinergics, digitalis derivatives, antimalarial drugs, corticosteroids, erectile dysfunction drugs, phenothiazines, chlorpromazine, indomethacin and others [2]. ^e^ Detailed guidelines for the management of chronic multisymptom illness [48]. ^f^ Detailed guidelines for the management of mental health/behavioral conditions [49]. mTBI: Mild traumatic brain injury

Symptoms	Recommended Conventional Treatments
Physical: Post-Traumatic Headache (PTH)	Provide education on headache-stimulus control, use of stimulants, sleep hygiene, dietary modification, relaxation techniques, and physical therapy [2]. Follow headache guidelines provided by the United States Veterans Affairs and the Department of Defense^a^. Empiric, nonspecific pharmacologic treatment for post-traumatic headache includes over-the-counter analgesics (i.e., acetaminophen); avoid non-steroidal anti-inflammatory drugs for 24 hours post-injury to limit bleeding risk [31].Common migraine-specific pharmacologic treatments include triptans, ergotamines, and dihydroergotamines [31]. Common post-traumatic headache prophylaxis include treatment with anticonvulsants (i.e., Gabapentin, Topiramate), tricyclic antidepressants, beta-blockers, and calcium channel blockers [32, 33]. Anti-calcitonin gene-related peptide (CGRP) monoclonal antibodies (i.e., erenumab) may be useful in treating post-traumatic headache [34,35].
Physical: Dizziness/Disequilibrium	Rule out other potential causes (i.e., vertebral basilar insufficiency, orthostatic hypotension, polypharmacy), then refer to vestibular and balance rehabilitation therapy [2,36,37]. Patient education: minimize alcohol/caffeine intake; encourage sleep hygiene and physical activity; inform of fall-prevention techniques; prescribe antiemetics for nausea [36]. Vestibular suppressant medications (i.e., meclizine, scopolamine, and dimenhydrinate) should be avoided but may be effective in severe cases [2,38].
Physical: Sleep Disturbance^b^	Cognitive-behavioral therapy for Insomnia (CBTi) may be effective [2,39]. Provide education on diet modification, physical activity, sleep hygiene, stimulus control, and use of stimulants [2]. Medications for sleep initiation and maintenance (i.e., trazodone, mirtazapine, tricyclic antidepressants) may be effective but should be limited to short-term use [2]. Avoid benzodiazepines due to the risk of dependency and potential worsening of cognition and executive function [2,40].
Physical: Tinnitus	Most cases resolve within one month following traumatic brain injury [2]. Currently no recommended treatments [2]. Repetitive Transcranial Magnetic Stimulation (rTMS) for tinnitus may be used in the future [41,42].
Physical: Visual Symptoms	Most visual disturbances^c ^improve within minutes to hours. Refer to a vision specialist (i.e., neuro-ophthalmologist). if symptoms persist or worsen [2]. Review patient’s medication list, as visual dysfunctions may be caused or worsened by certain medications^d^ [2].
Physical: Fatigue	Provide education regarding lifestyle factors such as diet, exercise, and sleep hygiene [2]. Cognitive Behavioral Therapy [2,43].
Physical: Persistent Pain	Rehabilitation therapies [2]. Follow the United States Veterans Affairs and the Department of Defense guidelines for the management of chronic multisymptom illness^e^ [2]. Avoid opioid medications[2].
Cognitive: Problems with Memory, Attention, Executive Function	Referral to a cognitive rehabilitation therapist with expertise in traumatic brain injury rehabilitation [2,44]. Assistive devices and cognitive assist technologies for self-management, self-advocacy, and health monitoring may be effective [2]. Emphasize self-efficacy to avoid a “sick role” [2]. Amantadine hydrochloride may improve cognitive recovery following mTBI, but further research is needed [45].
Behavioral: Post-traumatic Stress Disorder, Major Depressive Disorder, Substance Misuse, Anxiety, and Mood Disorders	Follow the United States Veterans Affairs and the Department of Defense evidence-based mental health guidelines for each specific disorder^f ^[2].

An osteopathic approach to the treatment of TBI

Acute and chronic TBI can cause somatic dysfunction involving cranial and extracranial structures. Post-concussive syndrome (PCS) is a term used to describe the array of symptoms that commonly occur after traumatic brain injury. Such symptoms are described above in Table 2. The application of OMT has demonstrated efficacy in alleviating symptoms of PCS. In some cases, OMT may provide complete resolution of symptoms [50,51]. When applied early and frequently, OMT improves prognosis in the management of PCS [51].

A TBI case series described eleven Canadian male football players with a confirmed diagnosis of TBI demonstrated that techniques such as craniosacral therapy (CST), visceral manipulation (VM), and neural manipulation (NM) significantly improved symptoms of PCS. The following measures were tested after the application of OMT: pain rating scale scores (P = 0.0448), cervicogenic pain levels decreased (P = 0.0486), Dynavision average reaction time (P = 0.0332), memory test (P = 0.0156) scores, and cervical range of motion scores (P = 0.0377) [52]. Osteopathic manipulative treatment is a useful diagnostic and therapeutic tool that should be considered as a part of the treatment protocol for TBI.

In another study conducted on soldiers with a known history of TBI, King et. al, demonstrated that light touch manual treatment (LTMT) improved symptoms of headache, anxiety, PTSD, and pain. The study was completed on 10 soldiers between the ages of 27 and 45 years with a diagnosis of PTSD and who self-reported injury to the head at least two years before the start of the study. Ninety percent had a diagnosis of headache and 80% had a diagnosis of TBI. They concluded that self-reported anxiety and headache were decreased after two LTMT sessions (P=0.008-0.031). Also, pain and PTSD symptoms were decreased, with P-values equal to 0.039 and 0.013, respectively [53].

McCallister et. al. provided two cases of severe acute TBI that were treated with OMT during their hospital stay at a Level 1 regional trauma center. Both patients presented with somatic dysfunction which improved upon discharge. Patient 1 and Patient 2 had a GCS score of 7 and 14 on initial evaluation, respectively. Upon discharge, both patients had a GCS score of 15. The techniques used were ligamentous tension and myofascial release with a focus on the cranium, cervical spine, and diaphragm [8].

Additionally, techniques such as pedal pump and thoracic pump have been shown to reduce intracranial pressure in patients with TBI, albeit not significantly. Cramer et. al. studied the effect of the techniques on intracranial pressure (ICP) and cerebral perfusion pressure (CPP) in 24 comatose patients (15 men, 9 women) between the ages of 18 to 69 years. Patients were assigned to two groups. Group 1 had a baseline ICP greater than 20mmHg and group 2 had a baseline ICP less than 20mmHg. Both groups received 50 sessions of OMT in total (19 treatments for group 1 and 31 treatments for group 2). In group 1 patients, the pedal pump and thoracic pump decreased mean ICP by 0.58mmHg (P=0.254) and increased CPP by a mean value of 1.16 mm Hg (P=0.413). In group 2, the pedal pump and thoracic pump reduced ICP by 0.89 mmHg (P=0.254) and increased CPP mean value by 2.21 mmHg (P=0.245). The reduction in ICP after the application of the pedal pump and thoracic pump is not statistically significant in either group. However, no adverse effects were associated with these techniques. Additionally, these techniques were found to have no detrimental effects on patients. Rather, the human contact component of these techniques and the increase in lymphatic drainage may have contributed to recovery [54]. 

Conventional therapies have been implemented to treat TBI, yet many have failed to show promising improvement. Due to mechanisms of action limited to specific treatment targets, conventional treatment does not address the wide-ranging pathophysiology present in TBI. As such, clinical management of TBI offers a special opportunity for osteopathic physicians via osteopathic manipulative medicine. Further OMT techniques such as sinus drainage, occipito-atlantal (OA) junction release, and compression of the fourth ventricle (CV4) may be used as a potential adjunctive therapy to reduce inflammation in the brain. Such techniques increase lymphatic drainage and normalize cerebrospinal fluid production and flow [8,50].

Discussion 

Traumatic brain injury is complex neuropathology with unique considerations in the combat veteran population. Current practice guidelines recognize and mitigate presenting symptoms of TBI: headache, mood, and sleep disturbances. However, there is a critical gap in delineated effective treatment for the secondary injury cascade that occurs after the initial TBI injury, such as oxidative stress, increased excitotoxicity, and increased neuroinflammation. 

Combat veterans often present with grave comorbidities such as neuropsychiatric disorders, PTSD, suicide, alcohol disorders, and somatic dysfunctions (as seen above in Table 2). These conditions must be considered from a structural and functional perspective. An osteopathic approach to the management of TBI involves a deeper analysis of anatomy, structure, function, comorbid conditions, patient preferences, nutrition, and environmental factors. Such an integrative approach has promising potential for vulnerable populations, especially combat veterans.

Osteopathic manipulative treatment is effective in regulating the autonomic nervous system (ANS). The ANS dysregulation is a contributing factor to TBI symptomatology [55]. The frontal cortex regulates the vagal tone and myogenic tone [55]. Additionally, the amygdala modulates autonomic, endocrine, and cardiovascular responses [55]. During a closed head injury, the frontal cortex and the amygdala are prone to damage. Consequently, multisystem dysfunction may occur after a TBI due to dysregulation of the ANS and release of catecholamines. Therefore, patients with TBI who experience symptoms of ANS dysregulation may benefit from the application of OMT [56].

The pathophysiology of TBI is complex and multifactorial. One of the mechanisms involves the release of inflammatory cytokines in the brain. Persistent inflammatory cytokines in the brain can cause neurodegenerative diseases. Osteopathic manipulative treatment has been shown to reduce inflammatory cytokines in musculoskeletal conditions and improve disrupted homeostatic mechanisms [57]. Likewise, OMT poses a low risk and improves ANS dysregulation, making it a viable and appropriate option, especially in veterans with comorbid PTSD and other emotional disturbances.

Additionally, osteopathic medicine widely recognizes the critical importance of glymphatic drainage in the maintenance of homeostatic processes. As noted, the glymphatic system plays a crucial role in the clearance of toxic metabolites from the brain. Therefore, disruption of the glymphatic system may cause deleterious damage to the brain following a TBI. As such, the glymphatic drainage in the brain is a critical mainstay in the osteopathic approach to traumatic brain injury. A recent study by Kashyap et al. demonstrated that OMT in addition to standardized therapies helps improve cerebrospinal fluid (CSF) flow which is postulated to be a consequence of the improvement in the function of the glymphatic system [58]. Further research is required to improve TBI identification, diagnosis, and prognosis. Accordingly, additional investigations are warranted to optimize a treatment protocol tailored to the unique needs of combat veterans [58]. 

## Conclusions

As a "signature injury" of the Iraq and Afghanistan wars, TBI ranks among the most common service injuries in combat veterans. Many cases of mild TBI remain undetected and may lead to long-term adverse comorbidities such as PTSD, pain disorders, alcohol disorders, and psychiatric diagnoses. Veterans with TBI are almost twice as likely to die from suicide, in comparison to veterans without a history of TBI. Veterans diagnosed with TBI experience significant comorbid conditions and thus advocacy for improved care is justified and necessary. Despite revised VA/DoD guidelines for the management of TBI, mild cases remain undetected and underdiagnosed. Current conventional TBI treatments include over-the-counter (OTC) analgesics, migraine medications, cognitive behavioral therapy (CBT), anticonvulsants, vestibular suppressants, and the adoption of a healthy lifestyle. The standard of care targets neurological symptoms including headache, blurry vision, auditory disturbances, psychiatric conditions, and dizziness.

While conventional treatments may alleviate TBI-related symptoms, such treatments do not prevent the progression of brain damage due to inflammatory processes and increased ICP. Likewise, conventional therapies do not target the somatic dysfunctions associated with ANS dysregulation following TBI. On a greater scale, the current standard of care does not reduce serious comorbidities associated with TBI. The OMT treatments, such as neural manipulation and myofascial release, have demonstrated positive outcomes among individuals with TBI. Osteopathic manipulative therapies target ANS dysregulation, glymphatic drainage, disrupted homeostasis, and inflammation. Improvement in these physiological mechanisms may improve TBI prognosis and reduce the risk of comorbid conditions. Osteopathic manipulative therapy safely optimizes healing and poses a low risk of adverse events. Thus, OMT should be considered in the treatment of combat veterans with TBI and associated service-connected ANS dysregulation. Given the high rates of TBI-related suicides among combat veterans, greater awareness and advocacy are necessary to improve the quality of care. A personalized, whole-person approach is highly recommended when treating this honorably-deserving population.
